# Chilling and Freezing Temperature Stress Differently Influence Glucosinolates Content in *Brassica oleracea* var. *acephala*

**DOI:** 10.3390/plants10071305

**Published:** 2021-06-27

**Authors:** Valentina Ljubej, Ivana Radojčić Redovniković, Branka Salopek-Sondi, Ana Smolko, Sanja Roje, Dunja Šamec

**Affiliations:** 1 Department of Molecular Biology, Ruđer Bošković Institute, Bijenička cesta 54, 10000 Zagreb, Croatia; kruk.valentina@gmail.com (V.L.); salopek@irb.hr (B.S.-S.); ana.smolko@irb.hr (A.S.); 2 Faculty of Food Technology and Biotechnology, University of Zagreb, Pierottijeva 6, 10000 Zagreb, Croatia; irredovnikovic@pbf.hr; 3 Institute of Biological Chemistry, Washington State University, Pullman, WA 99164, USA; sanja@wsu.edu; 4 Department of Food Technology, University North, University Center Koprivnica, Trg dr. Žarka Dolinara 1, 48000 Koprivnica, Croatia

**Keywords:** *Brassica oleracea* var. *acephala*, low temperature stress, glucosinolates, polyphenols, abiotic stress

## Abstract

*Brassica oleracea* var. *acephala* is known to have a strong tolerance to low temperatures, but the protective mechanisms enabling this tolerance are unknown. Simultaneously, this species is rich in health-promoting compounds such as polyphenols, carotenoids, and glucosinolates. We hypothesize that these metabolites play an important role in the ability to adapt to low temperature stress. To test this hypothesis, we exposed plants to chilling (8 °C) and additional freezing (−8 °C) temperatures under controlled laboratory conditions and determined the levels of proline, chlorophylls, carotenoids, polyphenols, and glucosinolates. Compared with that of the control (21 °C), the chilling and freezing temperatures increased the contents of proline, phenolic acids, and flavonoids. Detailed analysis of individual glucosinolates showed that chilling increased the total amount of aliphatic glucosinolates, while freezing increased the total amount of indolic glucosinolates, including the most abundant indolic glucosinolate glucobrassicin. Our data suggest that glucosinolates are involved in protection against low temperature stress. Individual glucosinolate species are likely to be involved in different protective mechanisms because they show different accumulation trends at chilling and freezing temperatures.

## 1. Introduction

Vegetables from the *Brassica oleracea* subgroup *Acephala* originated in the Mediterranean region but gained popularity worldwide [1]. This group of vegetables is characterized by leaves that do not form a head. Like other *Brassica* vegetables, they are considered a functional food due to the scientific evidence linking their consumption to numerous health benefits [2,3]. The health-promoting activities are associated with the presence of glucosinolates, polyphenols, carotenoids, various vitamins, minerals, dietary fibers etc. [4,5]. Due to their low caloric value but high content of fiber and phytochemicals, these vegetables are particularly popular among people who want to follow healthy dietary patterns [6].

The popularity of *Brassica* vegetables from the *Acephala* group may be related to their strong tolerance to unfavorable environmental conditions, which makes them an excellent crop for cultivation in the current period of climate change [1]. Under increasing environmental stress, the cellular and physiological processes become more and more compromised until limiting conditions for survival are reached. Plants respond to stress conditions with changes in the expression pattern of genes encoding proteins that control the biosynthesis of metabolites involved in the interactions between a given plant and its environment. This is an effort by plants to maximize their chances of survival under stress and maintain cellular function by synthesizing basic metabolites required for survival (primary metabolism) and specialized metabolites for specific environmental interactions (specialized metabolism). Specialized metabolites play critical roles in various physiological and pathological processes by participating in biochemical reactions required for proper biological function [7,8].

Our previous studies directly compared the tolerance to drought and salt stress of several *Brassica* species and showed that kale has the best stress tolerance in the group, correlating with higher levels of specialized metabolites, particularly phenolic compounds [8,9,10,11]. Low-temperature stress was also shown to influence specialized metabolism in *Brassica* plants. For example, flavonoids and anthocyanins were reported to accumulate in response to cold and frost stress in *Brassica rapa* [12,13,14], while low temperature stress leads to accumulation of specific flavonol glycosides and hydroxycinnamic acid derivatives in *Brassica oleracea* var. *sabellica* [15]. In addition to phenolic compounds, low-temperature regimes may be associated with higher carotenoid accumulation [16]. Glucosinolates play an important role in the abiotic stress response, and their role and that of their degradation products in temperature stress is still under investigation [17].

Plant response to abiotic stress may involve the induction of biosynthesis of various metabolites (polyphenolic compounds, carotenoids, glucosinolates) that are beneficial to human health. Kale (*B. oleracea* var. *acephala)* is tolerant to low temperatures, and in culinary practice is considered to have the best organoleptic properties when harvested late in the growth season, after exposure to low temperatures and frost [18]. Study by Steindal et al. [18] showed that cold acclimation of kale increases soluble sugar content, thereby improving taste while decreasing unsaturated fatty acid and glucosinolate contents. Similarly, Jurkow et al. [19] showed that medium (−5.0 °C) and heavy frost (−15.0 °C) increased soluble sugar content, phenolics, and antioxidant activity of kale, which makes harvesting of kale leaves more desirable after exposure to low temperatures.

Considering the excellent cold tolerance of kale and the high content of the above-mentioned health-promoting compounds (phenolic compounds, carotenoids, glucosinolates), we first investigated their content in kale under low temperature stress. Then we focused particularly on the analysis of selected indolic and aliphatic glucosinolates. Some previous studies showed that the content of health-promoting compounds may change at low temperatures, but most of these studies were conducted in the field, where other environmental factors are difficult to control. In the present study, we subjected kale to chilling and freezing temperature compared to the control under laboratory conditions where all other parameters expect temperature were constant. In this way, we minimized all other environmental effects that could affect changes in phytochemical levels. In the control plants (21 °C) and plants exposed to chilling (8 °C) and freezing (−8 °C) temperatures, we determined the levels of proline, polyphenolic compounds, carotenoids, chlorophylls, and total and individual glucosinolates to investigate their involvement in the response of kale to low temperature.

## 2. Results

### 2.1. Proline Content

**Figure 1:**
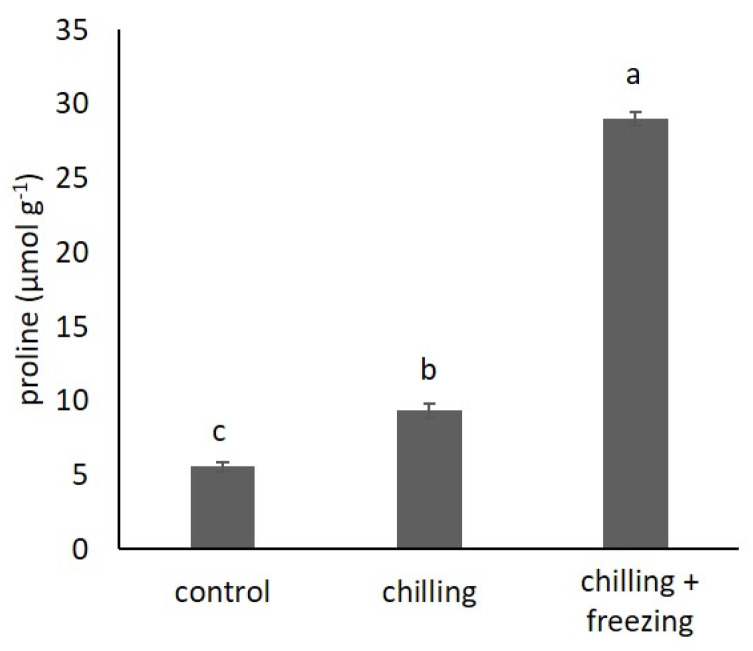
Proline content in *Brassica oleracea* var. *acephala* under control (21 °C), chilling (8 °C), and chilling (8 °C) + freezing (−8 °C) temperatures. Value marked with different letters are significantly different at *p* < 0.05.

Change in proline content in kale plants exposed to low temperatures is shown in Figure 1. The proline content in control samples was 5.40 ± 0.30 µmol g^−1^ and increased significantly under low temperatures. At chilling temperature, the proline content doubled, while at freezing temperature, the proline content increased 5-fold compared to the control.

### 2.2. Contents of Chlorophylls and Carotenoids

**Figure 2:**
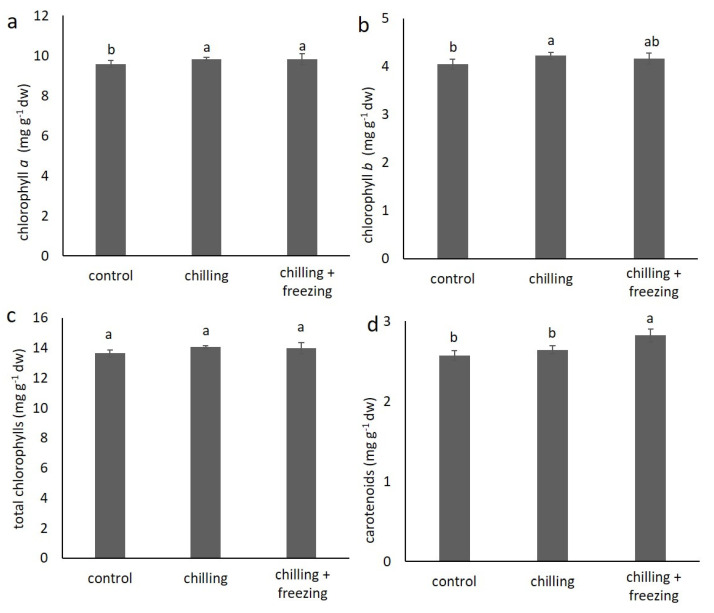
Content of chlorophyll *a* (**a**), chlorophyll *b* (**b**), total chlorophylls (**c**), and carotenoids (**d**) in *Brassica oleracea* var. *acephala* under control (21 °C), chilling (8 °C), and chilling (8 °C) + freezing (−8 °C) temperatures. Values marked with different letters are significantly different at *p* < 0.05.

The content of pigments, chlorophyll *a*, *b*, total chlorophylls, and carotenoids in *B.oleracea* var. *acephala* under chilling and freezing temperatures is shown in Figure 2. Chilling and freezing temperatures increased the content of chlorophyll *a* and chlorophyll *b* but total chlorophylls content statistically did not differ. Significant increase in carotenoid contents was seen only in the samples under freezing temperature.

The ratio of chlorophyll *a* and chlorophyll *b*, as well as the ratio of total chlorophylls and total carotenoids, may indicate some physiological processes in plants [20]. Therefore, we also evaluated these parameters (as illustrated in Table 1). The ratio of chlorophyll *a* to chlorophyll *b* was just above 2 in all three groups and did not differ significantly between treatments. The ratio of total chlorophylls to total carotenoids was comparable in the control and chilling groups, while it decreased significantly under the freezing temperature.

### 2.3. Polyphenolic Compound Contents

**Figure 3:**
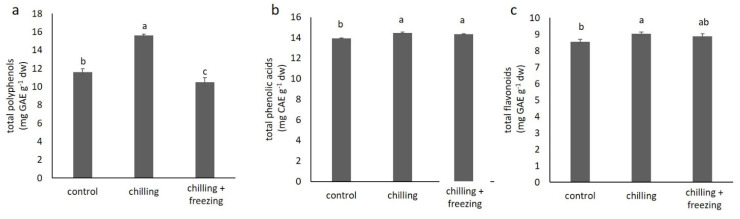
Total polyphenol (**a**), total phenolic acid (**b**), and total flavonoid (**c**) contents in *Brassica oleracea* var. *acephala* under control (21 °C), chilling (8 °C), and chilling (8 °C) + freezing (−8 °C) temperatures. Values marked with different letters are significantly different at *p* < 0.05.

The contents of total polyphenols, phenolic acids, and flavonoids under chilling and freezing temperature conditions compared to that of the control are shown in Figure 3. The total polyphenol content in the control samples was 11.61 ± 0.33 mg GAE g^−1^ dw; in samples under chilling temperature, it was significantly higher (15.60 ± 0.16 mg GAE g^−1^ dw), while under freezing temperature, it was slightly lower than in that of the control (10.51 ± 0.47 mg GAE g^−1^). The analysis of total flavonoids and phenolic acids shows that the increase in total polyphenolics under chilling temperature could be due to the increase in total phenolic acids and flavonoids (total phenolic acids: 14.47 ± 0.08 mg CAE g^−1^ dw; total flavonoids: 9.04 ± 0.10 mg CE g^−1^ dw), compared to that of the control (total phenolic acids: 13.95 ± 0.02 mg CAE g^−1^ dw; total flavonoids: 8.55 ± 0.14 mg CE g^−1^ dw). According to our results, freezing temperature led to increase in total phenolic acids and total flavonoids to 14.34 ± 0.06 mg CAE g^−1^ dw and 8.87 ± 0.17 mg CE g^−1^ dw respectively, but this increase was smaller than under the chilling treatment.

### 2.4. Glucosinolate Contents

**Figure 4:**
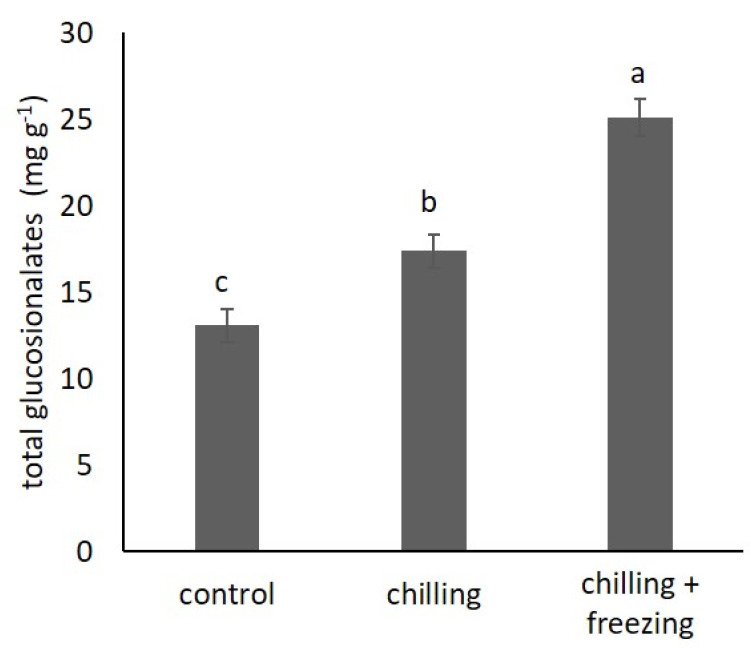
Spectrophotometrically measured total glucosinolate content in *Brassica oleracea* var. *acephala* under control (21 °C), chilling (8 °C), and chilling (8 °C) + freezing (−8 °C) temperatures. Values marked with different letters are significantly different at *p* < 0.05.

Glucosinolates are sulfur-containing phytochemicals found in cruciferous *Brassica* vegetables. In our study, we measured their content using UV/VIS spectrophotometer and HPLC-DAD. The results for total glucosinolate contents by spectrophotometric determination are shown in Figure 4. The results show a significant increase in the total content of glucosinolates with decreasing temperature. The total content of glucosinolates was 13.09 ± 0.97 mg g^−1^ dw in control, 17.39 ± 0.97 mg g^−1^ dw under chilling, and 25.12 ± 1.06 mg g^−1^ dw under freezing.

**Figure 5:**
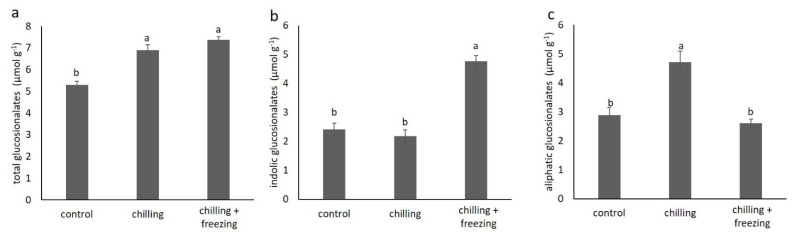
The content (in μmol g^−1^ dw) of total glucosinolates (**a**), indolic glucosinolates (**b**), and aliphatic glucosinolates (**c**) measured by HPLC-DAD in *Brassica oleracea* var. *acephala* under control (21 °C), chilling (8 °C) and chilling (8 °C) + freezing (−8 °C) temperatures. Values marked with different letters are significantly different at *p* < 0.05.

To analyze changes in individual glucosionalates in *B. oleracea* var. *acephala* under low temperature stress, we also analyzed glucosinolates by HPLC-DAD. In Figure 5, we show the content of total glucosionolates (a) and the content of total indolic (b) and aliphatic glucosinolates (c) measured by HPLC-DAD.

As can be seen from Figure 5, the HPLC-DAD measurement was in agreement with the spectrophotometric data showing that low temperature treatments increased the content of glucosinolates. However, the increase in the total content of glucosionaltes under freezing temperature as measured by HPLC-DAD was lower than what was measured spectrophotometrically. The HPLC-DAD measurement also showed that chilling temperature increased aliphatic glucosinolates while freezing temperature increased indolic glucosinolates. The content of individual glucosionolates is shown in Table 2.

In the control plants, indolic glucosionolate glucobrassicin was the most abundant glucosinolate species measured. Its content nearly doubled, while the content of the less abundant neoglucobrassicin also increased under the freezing treatment. These compounds probably contribute to the significant increase in indolic glucosionolates under the freezing conditions (as illustrated in Figure 5b). Glucobrassicin and 4-methoxyglucobrassicin content did not change under chilling or freezing treatment.

The most abundant aliphatic glucosionolate in the control samples was glucoiberin which increased by 50% under chilling temperature, but its content in the samples at freezing temperature was comparable to that of the corresponding control. The content of progoitrin and glucoraphanin was also increased significantly after chilling. For sinigrin, there is a clear trend of increase by lowering the temperature. It was increased 1.5-fold and 1.9-fold after chilling and freezing, respectively.

## 3. Discussion

Specialized metabolites play important roles in plant-environmental interactions, although their specific roles in plants are still not clear. Therefore, in our study we sought to determine the levels of bioactive compounds in *B. oleracea* var. *acephala* under low temperature stress to narrow down the individual species that may be involved in the interaction.

First, we determined the content of proline, which is a well-known stress marker [21]. Proline can act as an osmolyte, but it is also known for its role as a metal chelator, an antioxidant defense molecule, and a signaling molecule [21]. Thus, it is not surprising that we found a significant increase in proline content under chilling (1.7 -fold) and freezing (5.2-fold) conditions compared to that of the control. A similar trend of increase in proline contents at low temperatures was previously reported for *B. oleracea* var. *acephala* [22] and rapeseed (*Brassica napus* L.) [23,24].

Stressful conditions can reduce photosynthetic ability of plants because photosynthesis is an integrated and tightly regulated process that is very sensitive to any change in environmental conditions [25]. Low temperatures reportedly affect chlorophyll levels in plant species depending on their cold tolerance [25,26]. Plants with good cold tolerance maintain stable chlorophyll content, while plants with low cold tolerance experience a decrease in chlorophyll content [26]. Atici et al. [22] reported a decrease in chlorophyll content compared to that of the control in *B. oleracea* var *acephala* during acclimation to low temperatures in the first 15 days of exposure, while after 45 days, chlorophyll content was higher in plants grown under cold conditions than in that of the control. This might indicate adaptation of photosynthetic apparatuses to cold temperature. In our study, small changes in chlorophyll and carotenoid contents are consistent with the fact that *B. oleracea* var *acephala* is a plant with good cold tolerance. Moreover, the ratio of total chlorophyll/total carotenoids is an indicator of damage to the plant and the photosynthetic apparatus. Values below 4.2 are indicative of senescence, stress, and more rapid degradation of chlorophylls than carotenoids [27]. Although in our experiments values are not lower than 4.2, freezing temperature decreases this parameter, which may indicate reduced activity but not yet damage to the photosynthetic apparatus.

Polyphenols are a large group of specialized metabolites that play different roles in plant-environmental interactions (reviewed by Šamec et al. [8]). According to our results (as illustrated in Figure 3), chilling temperature significantly increases total polyphenol content, while freezing temperature decreases total polyphenol content compared to that of the control. According to the literature [1], polyphenolic compounds in *B. oleracea* var. *acephala* belong mainly to phenolic acids and flavonoids, which we measured in our experiment (as illustrated in Figure 3b,c). We found an increase in the content of phenolic acids and flavonoids under chilling and freezing temperatures, indicating the involvement of these compounds in stress tolerance at low temperatures. This is consistent with literature data for several *Brassica* species where authors reported an increase in flavonoids and/or phenolic acids under low temperatures [15,28]. Sharma et al. [29] summarized that low temperature stress in plants increases the expression of phenylpropanoid pathway enzymes such as PAL (phenylalanine ammonia lyase), CAD (cinnamylalcohol dehydrogenase), and HCT (hydroxycinnamoyl transferase) and consequently increases phenolic content. Increased phenolic levels further contribute to detoxification of ROS and accumulation of polyphenolic compounds in plant cell walls and increase cell wall thickness [8]. This process is beneficial for the prevention of chilling damage and cell collapse under cold stress [29].

Glucosinolates are a large group of specialized metabolites present in *Brassica* species. There is a growing body of scientific evidence showing that they are an important factor in abiotic stress responses [7,17]. Our spectrophotometric data showed significant increase in glucosinolate contents at lower temperatures (as illustrated in Figure 5). An interesting trend is observed for total indolic and aliphatic glucosinolates calculated based on HPLC-DAD analysis. Our data showed that chilling temperature increased aliphatic glucosinolates, while freezing temperature increased indolic glucosinolates. In contrast, Sarıkamış and Çakır [30] reported that low temperature (0 °C) significantly decreased both aliphatic and indolic glucosinolates in broccoli (*Brassica oleracea* var. *italic* L.), and they concluded that this was due to hydrolysis and degradation of glucosinolates during cell disruption, which could be the result of low temperature treatments. Similarly, Steindal et al. [18] reported that cold acclimation of *B. oleracea* var *acephala* generally resulted in some reduction in the content of total and individual glucosinolates. They reported that the most abundant glucosinolate in *B. oleracea* var *acephala* is glucobrassicin, which is consistent with our data. Each cruciferous species/variety shows a characteristic glucosinolate profile that includes more than ten different glucosinolates, although only 3–4 are typically predominant [31]. Glucobrassicin is reported as the predominant glucosinolate in *B. oleracea* var. *acephala* in several other studies [32,33]. According to our data, glucobrassicin content remains stable at chilling temperature but nearly doubles at freezing temperature. We found that the most abundant aliphatic glucosinolate in the control plants was glucoiberin, whose content increased under chilling temperature, while under freezing temperature it was comparable to that of the control plants. Only sinigrin showed a clear trend of increasing content with decreasing temperature. Our data suggest that glucosinolates are involved in low temperature stress, but also that individual glucosinolates show a different trend under chilling and freezing temperature. A similar trend was reported for broccoli [30]. We speculate that this is because different glucosinolate species are involved in different protective mechanisms due to different chemical structures and bioactivities.

## 4. Materials and Methods

### 4.1. Plant Growth and Low Temperature Treatment

**Figure 6:**
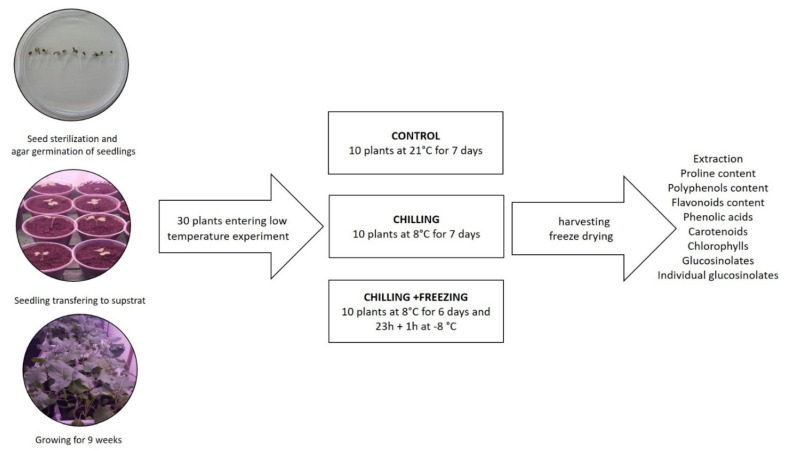
Scheme of the low temperature stress experiments.

Seeds were purchased from family farm Srđan Franić from Vrgorac, Croatia. Seed sterilization and germination were described previously [34]. Germinated seedlings were placed in a commercial substrate Stender A240 (Schermbeck, Germany) for plant growth and grown under controlled environmental conditions at a temperature of 21 °C and 16/8 h photoperiod (light/dark). After 4 weeks, all plants were repotted into larger pots with new substrate to ensure sufficient plant nutrients throughout the experiments. Each individual plant grew in a separate pot and was watered regulary. When the plants were 9 weeks old, 10 plants remained in a chamber at 21 °C serving as the control while 20 plants were transfered to a chamber where the temperature was set at 8 °C and remained there for 7 days under identical conditions of photoperiod and light intensity to the first group. Exactly after 6 days and 23 h at 8 °C, 10 plants were exposed furthermore to freezing temperature at the chamber set at −8 °C for one hour. Plants from all three temperature regimes were harvested at the same time, frozen in liquid nitrogen, and stored at −80 °C. All samples were then freeze-dried at the same time in Lyovac GT 2 (Steris GmbH, Köln, Germany). The freeze-dried samples were used for further extraction and analysis. The sheme of the low temperature experiments is shown in Figure 6.

### 4.2. Determination of Proline Content

Proline content was determined using 1% ninhydrin as we reported earlier [10]. For extraction, we used 30 mg of the freeze-dried tissue and 70% ethanol. Then, 100 μL of the obtained extract were mixed with 1 mL of reaction mixture containing 1% ninhydrin, 60% acetic acid, and 20% ethanol. Mixtures were heated at 95 °C for 20 min, cooled, and proline levels were measured at 520 nm using a UV–VIS spectrophotometer (Shimadzu BioSpec-1601, Kyoto, Japan). For the calibration curve proline standard (Sigma–Aldrich, Saint Louis, MO, USA) was used (0–1.6 mM, y = 1.4331x, R^2^ = 0.999) and results are expressed as μmol g^−1^ dw.

### 4.3. Determination of Total Phenol Content

For the determination of total phenols, we prepared an extract from 60 mg of dry samples and 2 mL of 80% methanol and determined total phenols using Folin–Ciocalteu reagent, as previously reported [35]. For the calibration curve, we used gallic acid (Sigma–Aldrich, USA) (0–1 g/mL, y = 0.001x, R^2^ = 0.999) and the results are expressed as gallic acid equivalents per dry weight mg GAE g^−1^ dw.

### 4.4. Determination of Total Phenolic Acids

Total phenolic acids were determined using Arnow’s reagent (10 g sodium nitrite, 10 g sodium molybdate, and distilled water up to 100 mL) [36]. The method was adopted for a small volume as follows. In brief, 300 µL of distillated water, 300 µL of the plant extract, 100 µL of Arnow’s reagent and 100 µL of 0.5 M hydrochloric acid per sample were mixed together. Next, 100 µL of 1 M sodium hydroxide and 100 µL of distilled water were added. Mixure were centrifugeted and absorbance was measured at 490 nm. Caffeic acid (Sigma–Aldrich, USA) served as a standard for the construction of the calibrattion curve and the results were expresed as caffeic acid equivalents per dry weight (mg CAE g^−1^ dw).

### 4.5. Determination of Total Flavonoids

Total flavonoids were determined using the method with Al_2_Cl_3_ according to Zhinshen et al. [37], which we previously optimized for *Brassica* samples [35]. For the calibration curve, we used catechin (Sigma–Aldrich, USA) and results are expressed as catechin equivalents per dry weight (mg CE g^−1^ dw).

### 4.6. Determination of Chlorophyll and Carotenoid Contents

Contents of chlorophyll *a*, chlorophyll *b* and carotenoids were determined spectrophotometrically according Lichtenthaler et al. [20] in samples extracted with 80% acetone. Results were expresses as μg g^−1^ dw.

### 4.7. Determination of Total and Individual Glucosinolates

For spectrophotometric determination, we used the method reported by Aghajanzaden et al. [38] and optimized for kale samples [35]. For the calibration curve, we used sinigrin (Carl Roth, Karlsruhe, Germany) and the results were expressed as milligrams of sinigrin equivalent per gram of dry weight (mg sin g^−1^ dw).

Individual glucosinolates were determined and quantfied by HPLC-DAD using the optimized ISO method 10633-1 (1995) as we previously reported [39]. Results are expressed as μmol g^−1^ dw (dry weight).

### 4.8. Statistical Analysis

All analyses were performed in at least three replicates and results are expressed as a mean ± standard deviation (SD). All statistical analyses were performed using Microsoft Office Excel 2010 upgraded with XLSTAT (ver. 1 May 2011). One-way ANOVA and post hoc multiple mean comparison (Tukey’s HSD test) were performed and differences between measurements were considered significant at *p* < 0.05.

## 5. Conclusions

In our study, we determined the content of proline, chlorophylls, carotenoids, polyphenols, and glucosinolates in *B. oleracea* var. *acephala* plants subjected to chilling and freezing temperature stress under controlled laboratory conditions. Chilling and freezing significantly increase proline contents, confirming the stress status of the treated plants. Low temperature also causes the increase in the contents of chlorophylls, phenolic acids, flavonoids, and glucosinolates. Analysis of individual glucosinolates showed that chilling temperature increased aliphatic glucosinolates, while freezing temperature increased indolic glucosinolates. The most abundant glucosinolate was glucobrassicin, which increased significantly under freezing temperature. Our data suggest that glucosinolates play a role in low temperature stress, but that individual glucosinolates show different accumulation trends under chilling and freezing conditions. Further studies are needed that directly link the chemical structure of glucosinolates to their response to cold stress.

## Figures and Tables

**Table 1:** Ratio of chlorophyll *a*/chlorophyll *b* and total chlorophylls/total carotenoids in *Brassica oleracea* var. *acephala* under control (21 °C), chilling (8 °C), and chilling (8 °C) + freezing (−8 °C) temperatures. Values marked with different letters across column are significantly different at *p* < 0.05.

	Chlorophyll *a*/ Chlorophyll *b*	Total Chlorophylls/ Total Carotenoids
control	2.37 ± 0.06 ^a^	5.31 ± 0.07 ^a^
chilling	2.33 ± 0.04 ^a^	5.32 ± 0.08 ^a^
chilling + freezing	2.36 ± 0.03 ^a^	4.96 ± 0.03 ^b^

**Table 2:** The content of individual glucosinolates (μmol g^−1^ dw) measured by HPLC-DAD in *Brassica oleracea* var. *acephala* under control (21 °C), chilling (8 °C), and chilling (8 °C) + freezing (−8 °C) temperatures. Values marked with different letters across rows are significantly different at *p* < 0.05.

		Control	Chilling	Chilling + Freezing
aliphatic	Glucoiberin	1.87 ± 0.02 ^b^	2.75 ± 0.18 ^a^	1.13 ± 0.16 ^b^
	Progoitrin	0.19 ± 0.06 ^b^	0.32 ± 0.04 ^a^	nd
	Sinigrin	0.57 ± 0.06 ^c^	0.82 ± 0.12 ^b^	1.18 ± 0.10 ^a^
	Glucoraphanin	0.25 ± 0.01 ^c^	0.85 ± 0.06 ^a^	0.41 ± 0.05 ^b^
	Gluconapin	nd	nd	nd
indolic	4-hydroxyglucobrassicin	0.18 ± 0.02 ^a^	0.20 ± 0.05 ^a^	0.15 ± 0.01 ^a^
	Glucobrassicin	2.05 ± 0.36 ^b^	1.86 ± 0.16 ^b^	3.98 ± 0.18 ^a^
	4-methoxyglucobrassicin	0.10 ± 0.01 ^a^	0.05 ± 0.02 ^a^	0.07 ± 0.06 ^a^
	Neoglucobrasscin	0.09 ± 0.03 ^b^	0.04 ± 0.01 ^c^	0.14 ± 0.02 ^a^

## Data Availability

Additional data are available upon request.
